# Neurological outcome and mortality in polytrauma patients with severe traumatic brain injury and massive transfusion protocol

**DOI:** 10.1590/1806-9282.20251907

**Published:** 2026-09-21

**Authors:** Maurício Godinho, Marcelo Bellini Dalio, Sandro Scarpelini, Edwaldo Edner Joviliano

**Affiliations:** 1 Universidade de São Paulo, Ribeirão Preto Medical School, Department of Surgery and Anatomy, Division of Urgence Surgery – Ribeirão Preto (SP), Brazil.; 2 Universidade de São Paulo, Ribeirão Preto Medical School, Department of Surgery and Anatomy, Division of Vascular and Endovascular Surgery – Ribeirão Preto (SP), Brazil.

**Keywords:** Closed head injury, Brain injuries, traumatic, Blood transfusion, Multiple trauma, Mortality

## Abstract

**OBJECTIVE:**

The aim of this study was to evaluate neurological outcomes and mortality in polytrauma patients with severe traumatic brain injury who were included in a massive transfusion protocol compared to those who were not.

**METHODS:**

Retrospective analysis of adult traffic accident with blunt head trauma and severe traumatic brain injury between 2013 and 2018. Inclusion criteria: Glasgow Coma Scale score≤8, Abbreviated Injury Scale score≥3, Injury Severity Score≥16. Patients were divided into two groups: those who received massive transfusion protocol (massive transfusion protocol group) and those who did not (No massive transfusion protocol group). Data collected included demographics, accident type, severity scores, serum lactate, and base excess. Neurological outcomes were assessed using the modified Rankin Scale. Mortality and hospital length of stay were recorded.

**RESULTS:**

77 patients met the inclusion criteria (massive transfusion protocol group: n=26 [33.8%]; No massive transfusion protocol group: n=51 [66.2%]). Most patients were young males involved in motorcycle accidents. Trauma scores, lactate, and base excess were similar between groups. The mean modified Rankin Scale score at discharge was lower in the massive transfusion protocol group (massive transfusion protocol: 4.65 vs. No massive transfusion protocol: 5.41; p=0.0313), indicating better neurological outcomes. Among surviving patients, the distribution of non-fatal modified Rankin Scale categories was similar between groups. Mortality (modified Rankin Scale 6) was significantly lower in the massive transfusion protocol group (57.7%) compared to the No massive transfusion protocol group (80.4%; p=0.007). Hospital length of stay in days was similar in both groups.

**CONCLUSION:**

Inclusion in a massive transfusion protocol was associated with improved neurological outcomes and lower mortality in polytrauma patients with severe traumatic brain injury.

## INTRODUCTION

Traumatic brain injury (TBI) and massive blood loss co-occur in polytrauma, contributing to high morbidity and mortality rates^1^. Approximately 26% of patients with severe TBI also experience hemorrhagic shock, and half of the deaths occur early^2^. For decades, it has been recognized that the prognosis for polytrauma patients with both severe TBI and massive blood loss is worse than for those with isolated TBI^3^. However, an approach to resuscitation that does not exacerbate neurological injury has yet to be standardized.

Massive transfusion protocols (MTPs) assist trauma teams and transfusion services in managing such critical cases^4^. In addition to ensuring the immediate availability of blood components, MTPs provide guidance on the appropriate types and ratios of blood products to transfuse. They also define the responsibilities of each healthcare professional involved in the transfusion process^5^. Studies have shown that MTPs can reduce morbidity and mortality by ensuring that blood components are administered only when indicated. These indications may be based on clinical findings, laboratory parameters, or activation scoring systems^5^.

There is limited discussion regarding the management of TBI in polytrauma when combined with injuries to other organ systems - particularly in the context of MTP activation for patients in severe hemorrhagic shock^6^. Most studies focused exclusively on isolated TBI and found no benefit in the liberal use of blood products. These liberal transfusion strategies have not improved patient outcomes and may be detrimental in cases of severe injury^6,7^. Recently, restrictive transfusion strategies, such as maintaining hemoglobin levels at a minimum of 7 g/dL, have been adopted by neurosurgeons to reduce secondary brain injury^7^.

Ten years after the implementation of an MTP in our hospital, we aimed to evaluate the outcomes of this intervention in polytrauma patients with severe TBI. This study was designed to assess neurological outcomes and mortality in patients with severe TBI who were included in a MTP compared to those who were not.

## METHODS

### Study design

We conducted a retrospective analysis of polytrauma patients with severe TBI. Patients who were included in the MTP group and received liberal transfusions were compared with those who were not included in the protocol (No MTP group). The decision to activate the MTP was made at the discretion of the attending physician. The study was approved by the Institutional Ethics and Research Committee (approval number 3.304.655), in accordance with the Declaration of Helsinki and local ethical guidelines. The requirement for individual informed consent was waived.

### Setting

This study was conducted in the emergency unit of our hospital, a tertiary referral center for acute trauma and medical or surgical emergencies.

### Population

We reviewed the medical records of all adult patients (aged >15 years) involved in traffic accidents who sustained blunt head trauma and severe TBI between August 2013 and December 2018. Patients aged <15 years were not included because, at our institution, they are treated in the pediatric trauma service under different resuscitation and transfusion protocols. Inclusion criteria: Glasgow Coma Scale (GCS) score≤8, Abbreviated Injury Scale (AIS) score≥3, Injury Severity Score (ISS)≥16. Patients who were declared dead on arrival were excluded.

### Massive transfusion protocol

The MTP is a multidisciplinary process designed to ensure the rapid delivery of blood and blood products to critically ill patients. It was available throughout the study period in our emergency unit. The protocol could be activated by the surgeon, anesthesiologist, or intensive care specialist in cases of massive blood loss. MTP activation was guided by a combination of the Assessment of Blood Consumption (ABC) score and the Shock Index (SI). According to the ABC score, the presence of two or more of the following criteria indicates the need for MTP activation: systolic blood pressure<90 mmHg, heart rate>120 bpm, penetrating mechanism of injury, and positive abdominal ultrasound (Focused Assessment with Sonography in Trauma [FAST]). The SI is calculated by dividing the heart rate by the systolic blood pressure: <0.6=no shock; 0.6–1.0=minor shock; 1.0–1.4=moderate shock; ≥1.4=severe shock. MTP activation was recommended for SI>1, but the final decision rested with the attending physician. In cases where MTP was not activated, transfusions were prescribed individually by the physician.

Upon MTP activation, the transfusion service delivered Pack 1 (two type O packed red blood cell [pRBC] units and one type A plasma unit). This was followed by Pack 2: four ABO-compatible pRBC units and two ABO-compatible plasma units; Pack 3: same as Pack 2 plus 10 cryoprecipitate units; Pack 4: same as Pack 2 plus one platelet unit; and Pack 5: same as Pack 2. Additionally, patients received 1 g of tranexamic acid intravenously over 10 min, followed by 1 g for the next 8 h.

Progression through the sequential transfusion packs was guided by persistent evidence of hemorrhagic shock and ongoing bleeding, including hemodynamic instability, ongoing transfusion requirements, vasopressor support, metabolic acidosis, elevated lactate levels, and the attending physician's clinical judgment. The protocol was discontinued after surgical or clinical control of bleeding and sustained hemodynamic stabilization without evidence of ongoing major hemorrhage.

### Collected data and outcomes

Data collected included demographics, type of traffic accident, and admission values for GCS, ISS, Revised Trauma Score (RTS), and Trauma and Injury Severity Score (TRISS). Serum lactate and base excess were recorded at admission.

Neurological outcomes were assessed using the modified Rankin Scale (mRS)^8^, evaluated at discharge or death. The mRS ranges from 0 to 6: 0=no symptoms; 1–2=mild disability; 3=moderate disability; 4–5=severe disability; 6=death.

An mRS score of 3 was considered the threshold between independence and disability. Thus, mRS scores≤2 were categorized as "independent," and scores≥3 as "disabled"^9^. Mortality and hospital length of stay were also recorded.

### Statistical analysis

Continuous variables were expressed as mean±standard deviation and compared between groups using the unpaired t-test. Categorical variables were presented as counts and percentages and compared using the chi-square test. mRS values were analyzed as both continuous and categorical variables. A p-value of <0.05 was considered statistically significant.

## RESULTS

Out of 519 traffic accident cases involving blunt head trauma and severe TBI, 77 met the inclusion criteria (GCS≤8, AIS≥3, and ISS≥16) and were included. Twenty-six patients (33.8%) were included in the MTP group and received liberal transfusions, while 51 patients (66.2%) were assigned to the No MTP group.

Table 1 presents the demographic characteristics, accident types, admission trauma scores, serum lactate, and base excess for both groups. Trauma severity scores, serum lactate, and base excess were consistent with a critically ill population, with no significant differences between the two groups.

**Table 1:** Demographic characteristics, type of accident and admission trauma scores and serum lactate and base excess values in all patients in massive transfusion protocol and No massive transfusion protocol groups.

		Total (n=77)	MTP (n=26)	No MTP (n=51)	p-value
Age (years), mean±SD		40.9±17.9	41.5±15.3	40.1±10.3	0.6352^a^
Male sex, n (%)		60 (77.9%)	21 (80.8%)	39 (76.5%)	0.6671^b^
Type of accident, n (%)					
	Motorcycle	34 (44.2%)	12 (46.2%)	22 (43.1%)	0.8010^b^
	Pedestrian hit by motor vehicle	21 (24.3%)	8 (30.8%)	13 (25.5%)	0.6228^b^
	Automobile	14 (18.2%)	5 (19.2%)	9 (17.6%)	0.8647^b^
	Bicycle	8 (10.4%)	1 (3.8%)	7 (13.7%)	0.1791^b^
Admission trauma scores, mean±SD					
	GCS	3.6±1.4	3.6±1.5	3.5±1.2	0.6851^a^
	ISS	28.8±9.5	29±9.1	27.9±10.2	0.6362^a^
	RTS	3.4±1.2	3.1±1.5	3.5±1.1	0.1874^a^
	TRISS	39.2±29.9	39.5±25.4	37.9±22.1	0.7790^a^
Admission laboratory values, mean±SD					
	Serum lactate, mg/dL	7.1±4.0	7.0±4.1	7.5±3.1	0.4970^a^
	Base excess, mEq/L	-9.2±5.1	-8.5±4.7	-9.8±5.6	0.3135^a^

MTP: Massive transfusion protocol; No MTP: no massive transfusion protocol; SD: standard deviation; GCS: Glasgow coma scale; ISS: injury severity score; RTS: revised trauma score; TRISS: trauma and injury severity scores.

a unpaired t-test;

b chi-square test.

Table 2 summarizes the neurological outcomes, mortality rates, and hospital length of stay. The mean mRS score at discharge was significantly lower in the MTP group compared to the No MTP group (MTP: 4.65 vs. No MTP: 5.41; p=0.0313), indicating better neurological outcomes in the MTP group. Among surviving patients, the distribution of non-fatal mRS categories was similar between groups. Mortality (mRS 6) was significantly lower in the MTP group (57.7%) compared to the No MTP group (80.4%; p=0.007). Hospital length of stay in days was similar in both groups.

**Table 2:** Neurological outcome, mortality, and hospital stay in all patients in massive transfusion protocol and No massive transfusion protocol groups.

		Total (n=77)	MTP (n=26)	No MTP (n=51)	p-value
Mean mRS at discharge (or death), mean±SD		5.2±1.5	4.7±1.8	5.4±1.3	0.0313^a^
Distribution of mRS at discharge (or death), n (%)					
	0	1 (1.3%)	1 (3.8%)	0 (0%)	0.1586^b^
	1–2	8 (10.4%)	4 (15.4%)	4 (7.8%)	0.3051^b^
	3	3 (3.9%)	1 (3.8%)	2 (3.9%)	0.9871^b^
	4–5	9 (11.7%)	5 (19.2%)	4 (7.8%)	0.1413^b^
	6	56 (72.7%)	15 (57.7%)	41 (80.4%)	0.0344^b^
Mortality, n (%)		56 (72.7%)	15 (57.7%)	41 (80.4%)	0.0070^b^
Hospital stay (days), mean±SD		16.5±5.1	18.1±4.9	15.7±6.1	0.0860^a^

MTP: Massive transfusion protocol; No MTP: no massive transfusion protocol; SD: standard deviation; mRS: modified Rankin scale.

a unpaired t-test;

b chi-square test.

## DISCUSSION

This study demonstrated that polytrauma patients with severe TBI who were managed under a MTP had lower mortality and better neurological outcomes at hospital discharge compared with patients who did not undergo protocolized transfusion management. These findings suggest that organized and early transfusion strategies may provide clinical benefits in this critically ill population.

MTPs have become an important component of modern trauma care because they facilitate rapid blood product delivery and coordinated resuscitation. Previous studies have shown that early balanced transfusion strategies may reduce mortality in patients with severe hemorrhage^10^. Initial investigations suggested improved survival with higher plasma-to-pRBC ratios^11^, although later randomized trials and meta-analyses reported conflicting results regarding the optimal composition of transfusion packs^12,13^.

The management of severe TBI associated with hemorrhagic shock remains particularly challenging. Cerebral hypoperfusion secondary to hypotension is a well-established predictor of poor neurological outcome^2^, and even brief episodes of systemic shock may worsen secondary brain injury. Consequently, aggressive hemorrhage control and maintenance of adequate cerebral perfusion pressure are essential in this setting^14,15^.

At our institution, MTP activation was guided by the ABC score and SI, combined with clinical judgment. Patients included in the protocol received standardized blood component packs, allowing rapid correction of hypovolemia and coagulopathy. Protocol progression depended on persistent evidence of hemorrhagic shock, whereas discontinuation occurred after hemorrhage control and hemodynamic stabilization.

Trauma severity scores confirmed that the population included in this study was critically ill. The mean ISS was elevated, and all patients presented with severe neurological impairment on admission. Lactate and base excess values were also compatible with significant tissue hypoperfusion. Importantly, these variables were similar between groups, suggesting comparable initial trauma severity. The ISS remains a robust indicator of overall trauma severity despite newer scoring systems^16^. Likewise, although the GCS has recognized limitations in evaluating consciousness, it continues to be fundamental for assessing TBI severity^17^.

In recent years, physiological markers such as lactate and base excess have become increasingly important in the acute assessment of trauma patients. Elevated lactate levels are associated with tissue hypoperfusion and worse outcomes in trauma^15^. However, Park et al.^18^ reported that lactate may have limited prognostic value in patients with TBI undergoing liberal transfusion strategies.

Neurological outcome was evaluated using the mRS. Although originally developed for stroke assessment, the mRS has been increasingly used to assess functional dependence after neurological injury^8^. Concerns remain regarding interobserver variability^19^, but structured application improves its reproducibility^8^. In the present study, patients managed with the MTP demonstrated lower mean mRS scores at discharge, suggesting better neurological recovery among survivors.

The literature regarding transfusion strategies in TBI remains controversial. Restrictive transfusion protocols have been increasingly adopted in neurocritical care because of concerns about transfusion-related complications^7^. The 2023 European guideline on major bleeding recommends repeated hemoglobin and hematocrit measurements during trauma resuscitation^15^, although no consensus exists regarding ideal transfusion thresholds in severe TBI^20^. McIntyre et al.^21^ found no mortality difference between liberal and restrictive transfusion strategies in patients with moderate or severe head injury. Similarly, Warner et al.^22^ observed worse functional outcomes among transfused patients without differences in mortality. In contrast, Gobatto et al.^23^ demonstrated improved neurological outcomes and lower mortality with liberal transfusion strategies. A recent systematic review and meta-analysis favored restrictive protocols but emphasized the heterogeneity of available studies and the need for further investigation^24^.

In our study, patients included in the MTP group had better neurological outcomes, as assessed by the mRS, and significantly lower mortality compared with those not included in the protocol. Although the role of MTPs in severe TBI remains inconclusive in the current literature^24^, our findings suggest that protocolized and early transfusion management may provide clinical benefits in polytrauma patients with severe TBI and hemorrhagic shock.

This study has limitations. Its retrospective design and relatively small sample size limit the strength of causal inferences. Additionally, information regarding previous comorbidities and chronic medication use was inconsistently documented in several medical records, which prevented reliable analysis of these variables. Another limitation is the use of the mRS, originally designed for stroke patients, as an outcome measure in trauma.

Despite these limitations, the study applied strict inclusion criteria and evaluated a homogeneous cohort of critically ill polytrauma patients with severe TBI. The findings support further prospective investigations evaluating the role of MTPs in this population.

## CONCLUSION

Inclusion in a MTP was associated with improved neurological outcomes and lower mortality in polytrauma patients with severe TBI.

## Data Availability

The datasets generated and/or analyzed during the current study are available from the corresponding author upon reasonable request.
